# Process optimization and modeling of Cd^2+^ biosorption onto the free and immobilized *Turbinaria ornata* using Box–Behnken experimental design

**DOI:** 10.1038/s41598-022-07288-z

**Published:** 2022-02-28

**Authors:** Mustafa A. Fawzy, Hadeer Darwish, Sarah Alharthi, Mayasar I. Al-Zaban, Ahmed Noureldeen, Sedky H. A. Hassan

**Affiliations:** 1 Biology Department, Faculty of Science, Taif University, P.O. Box 11099, Taif, 21944 Saudi Arabia; 2 Biotechnology Department, Faculty of Science, Taif University, P.O. Box 11099, Taif, 21944 Saudi Arabia; 3 Chemistry Department, Faculty of Science, Taif University, P.O. Box 11099, Taif, 21944 Saudi Arabia; 4 Biology Department, Faculty of Science, Princess Nourah bint Abdulrahman University, Riyadh, 11671 Saudi Arabia; 5 Department of Biology, College of Science, Sultan Qaboos University, 123 Muscat, Oman; 6 Department of Botany and Microbiology, Faculty of Science, New Valley University, El-Kharga, 72511 Egypt

**Keywords:** Plant sciences, Environmental sciences

## Abstract

The release of effluents containing cadmium ions into aquatic ecosystems is hazardous to humans and marine organisms. In the current investigation, biosorption of Cd^2+^ ions from aqueous solutions by freely suspended and immobilized *Turbinaria ornata* biomasses was studied. Compared to free cells (94.34%), the maximum Cd^2+^ removal efficiency reached 98.65% for immobilized cells obtained via Box–Behnken design under optimized conditions comprising algal doses of 5.04 g L^−1^ and 4.96 g L^−1^, pH values of 5.06 and 6.84, and initial cadmium concentrations of 25.2 mg L^−1^ and 26.19 mg L^−1^, respectively. Langmuir, Freundlich, and Temkin isotherm models were suitably applied, providing the best suit of data for free and immobilized cells, but the Dubinin–Radushkevich model only matched the immobilized algal biomass. The maximum biosorption capacity of Cd^2+^ ions increased with the immobilized cells (29.6 mg g^−1^) compared to free cells (23.9 mg g^−1^). The Cd^2+^ biosorption data obtained for both biomasses followed pseudo-second-order and Elovich kinetic models. In addition, the biosorption process is controlled by film diffusion followed by intra-particle diffusion. Cd^2+^ biosorption onto the free and immobilized biomasses was spontaneous, feasible, and endothermic in nature, according to the determined thermodynamic parameters. The algal biomass was further examined via SEM/EDX and FTIR before and after Cd^2+^ biosorption. SEM/EDX analysis revealed Cd^2+^ ion binding onto the algal surface. Additionally, FTIR analysis confirmed the presence of numerous functional groups (hydroxyl, carboxyl, amine, phosphate, etc.) participating in Cd^2+^ biosorption. This study verified that immobilized algal biomasses constitute a cost-effective and favorable biosorbent material for heavy metal removal from ecosystems.

## Introduction

Heavy metals are natural ecosystem elements acknowledged to exhibit a wide enough abundance and distribution to be biologically or environmentally important as harmful elements^1^. Heavy metals are classified into essential and nonessential categories^2^. Essential heavy metals are less harmful at low quantities and serve as coenzymes in biological processes. Nonessential heavy metals are very hazardous even at low levels and cause serious health problems in humans, such as neurological, hematopoietic, renal, reproductive, cardiovascular, respiratory and gastrointestinal issues^3^. Furthermore, heavy metals impact animals in addition to the soil microbiological balance, reducing their fertility^4^. Zinc, chromium, copper, cadmium, mercury, arsenic and lead are among the metals that can be harmful to humans and microorganisms.

Among these heavy metals, cadmium is an important heavy metal employed in industry, which pollutes the environment. Agricultural chemicals, including fertilizers and pesticides, as well as industrial processes, such as battery production, vehicle exhaust, smelting, mining, petroleum processing and plating processes, are the major sources of cadmium^5^. Cadmium is not biodegradable and poses serious health risks to humans. Cadmium mostly accumulates in the liver and kidney, and elevated levels have been linked to chronic kidney disease^6^.

As a result, the need for less expensive, safe, and more effective methods to remove heavy metals from wastewater has necessitated research into low-cost alternatives to currently existing techniques^7^. Numerous approaches have been considered for the removal of toxic heavy metals from wastewater, such as solvent extraction, reverse osmosis, adsorption, ion exchange, and chemical precipitation^8,9^.

Biosorption can constitute a potential alternative strategy for the removal of heavy metals because this approach provides many advantages over conventional treatment techniques, such as environmental friendliness, high efficiency of metal binding, cost-effectiveness, high quality of diluted effluents, and biosorbent regeneration with metal recovery^10^. Biosorption utilizes low-cost biosorbents such as grape shoots, olive roots, peat, rose waste, bacteria, yeasts, fungi, microalgae and marine macroalgae for heavy metal elimination from wastewaters^11–13^. Algal biomass exhibits significant and various affinities toward a number of heavy metals as sorbent materials^14,15^.

The immobilization of algal biomass is an important phase during commercial scale-up of metal biosorption. Immobilization, in contrast to algal biomass in its natural state, provides biosorbent particles with the density, suitable size, and mechanical strength required by continuous systems. Immobilization can also save up to 60% of the overall biomass separation cost from the processed solution. Currently, alginate, chitosan, porous resin and activated charcoal have been tested as carriers for cell immobilization^16–19^. Alginate is a main constituent of the outer cell walls of brown algae and has been related to the higher metal absorption capacity of brown algae than that of other algae, fungi, and bacteria according to several investigations. Alginate is a linear copolymer of α-L-guluronate and β-D-mannuronate accounting for up to 10–40% of the dry weight of all brown algae species, and each constituent residue naturally comprises carboxyl groups^20^. The carboxylate functional groups of this polysaccharide are supposed to be responsible for its high affinity for divalent cations such as cadmium, copper, cobalt, and other heavy metals. The process of forming alginate beads (with an egg-box structure) by releasing a mixture comprising biosorbents and Na-alginate into a solution of calcium chloride has been widely studied. Calcium atoms can cross-link and form salt bridges between the guluronic acid blocks of alginate chain pairs. These pairs can dimerize with other pairs at the same time. Alternating free mannuronic acid cross-linked guluronic blocks comprise the polymer gel matrix^21^.

In light of the present state of heavy metal pollution of water bodies, it is necessary to investigate alternative adsorbents for metal removal. Until recently, the potential of employing modified native *Turbinaria ornata* biomass as a cost-effective biosorbent has remained of great interest, as this material has not been previously investigated regarding the biosorption of Cd^2+^ ions.

Since alginate exhibits a good biocompatibility, low cost, notable binding capacity and good hydrophilicity^22^, it was applied in this study as a carrier for marine brown macroalgae immobilization.

Enhancing the metal biosorption efficiency by the traditional approach is a costly, time-consuming, and laborious task. These drawbacks of the traditional approach can be overcome via the Box–Behnken design method, which decreases the number of tests and facilitates enhancement by examining the impact of individual and reciprocal interactions between factors on the response.

As a result, the current investigation aimed to optimize biosorption process factors such as the algal dose, pH, and initial metal concentration via Box–Behnken design application to Cd^2+^ removal from aqueous solutions by freely suspended and immobilized *Turbinaria ornata* biomasses. The metal biosorption efficiency of freely suspended and immobilized algal biomasses was quantitatively described and predicted with isotherm, kinetic, and thermodynamic models. The biomasses were further characterized before and after Cd^2+^ ion biosorption via FT-IR, SEM and EDX analysis.

## Results and discussion

### Effect of the initial Cd^2+^ ion concentration and contact time

Experiments were carried out at Cd^2+^ concentrations ranging from 10 to 100 mg L^−1^, biosorbent dosage of 4 g L^−1^, pH of 5, and contact time of 90 min to evaluate the maximum removal percentage of Cd^2+^ ions for free and immobilized algal cells.

**Figure 1:**
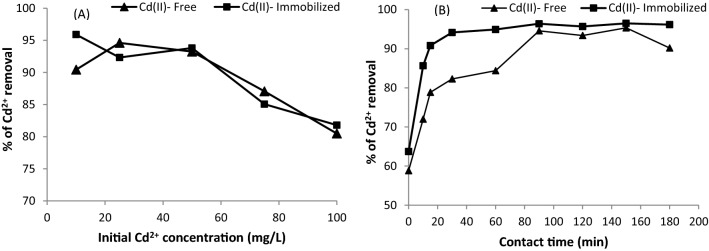
Impact of (**A**) the initial Cd^2+^ concentration and (**B**) contact time on the removal percentage of Cd^2+^ by the freely suspended and Ca-alginate-immobilized *T. ornata* cells (biosorbent dose = 4 g/L; pH = 5.0; contact time = 90 min; initial Cd^2+^ concentration = 10 mg/L; temperature 25 °C; shaking at 170 rpm).

As shown in Fig. 1A, the removal percentage of Cd^2+^ ions for the free biomass was enhanced until it reached 25 mg L^−1^, with a maximum removal percentage of 94.6%, before declining with increasing initial concentration of Cd^2+^. In contrast, the process of Cd^2+^ biosorption by the immobilized algal biomass decreased with increasing concentration of Cd^2+^, with the maximum removal percentage reaching 95.9% at a Cd^2+^ concentration of 10 mg L^−1^. At lower Cd^2+^ concentrations, more of the metal ions in the solution were associated with biosorption sites. Thus, Cd^2+^ ion biosorption rose gradually. However, with increasing Cd^2+^ concentration, biosorption decreased due to saturation of the available binding sites and a large number of Cd^2+^ ions vying for the remaining biosorption sites in the biosorbent material. A related finding was observed for the biosorption of copper ions onto *Codium vermilara*^15^.

The contact time significantly impacts the removal process of Cd^2+^ ions by free and immobilized algae (Fig. 1B). Under the conditions of an algal dosage of 4 g L^−1^, pH of 5 and initial Cd^2+^ concentration of 10 mg L^−1^, the effect of the contact time on the removal of Cd^2+^ ions was studied in the range from 0 to 180 min. The findings revealed that Cd^2+^ ion elimination was rapid during the first 15 min and then gradually improved until equilibrium was achieved at 90 min (94.6% and 96.4% for the free and immobilized *T. ornata* cells, respectively), in which the process of Cd^2+^ biosorption virtually stabilized. Since Cd^2+^ ions made contact with empty surface binding sites, Cd^2+^ biosorption was initially faster. However, once equilibrium was approached at 90 min, the biosorption sites were saturated, and no further biosorption likely occurred^23^. Davarnejad and Panahi^24^ demonstrated that with increasing contact time, the efficiency of removal decreased, which may be attributed to biosorption at all active sites in a monolayer. These findings also indicated that due to the surface biosorption effect of beads, the immobilized biomass of *T. ornata* could achieve high Cd^2+^ ion biosorption rates relatively fast.

### Statistical optimization of Cd^2+^ ion removal by freely suspended and immobilized T. ornata biomasses via Box–Behnken design (BBD)

The biosorption process is considered a complex system, and several factors affect the removal capacity, such as biosorbent and heavy metal concentrations and availability, as well as physicochemical variables, including the contact time, pH of the solution and temperature. The impact of some of these parameters on cadmium biosorption has been investigated.

According to the Box–Behnken design method, 17 tests were conducted with varying combinations of three factors (algal dosage, pH, and initial concentration of Cd^2+^) at three levels (coded and actual levels), including low (− 1), medium (0), and high (+ 1), for optimization of the Cd^2+^ removal conditions for maximum elimination of Cd^2+^ ions from aqueous solutions (Table S1).

Table 1 lists the actual and expected percentages of the Cd^2+^ ion removal efficiency for the 17 runs of the design matrix. The findings revealed that, based on the independent factors, the cadmium removal efficiency varies significantly with free and immobilized biomasses. According to the experimental results, the percentage of cadmium removed by the free algae varied between 70.48% and 92.62%, while the corresponding value for the immobilized biomass beads ranged from 73.08% to 95.87%.

**Table 1:** Results of ANOVA for the quadratic model coefficients of Cd^2+^ ions removal.

Model term	Coefficient estimate		Degrees of freedom		Standard error		*F* value		*p* value Prob > *F*	
	Free cells	Immob. cells	Free cells	Immob. cells	Free cells	Immob. cells	Free cells	Immob. cells	Free cells	Immob. cells
Intercept	87.3	86.8	1	1	1.12	1.48	–	–	–	–
A-algal dose	5.8	7.04	1	1	1.18	1.17	24.14	36.5	0.0005	0.0005
B-pH	2.8	2.8	1	1	1.18	1.17	5.62	5.7	0.0371	0.0478
C-initial Cd conc	− 4.8	− 5.2	1	1	1.18	1.17	16.50	19.9	0.0019	0.0029
AB	–	− 1.9	–	1	–	1.65	–	1.3	–	0.2982
AC	–	3.9	–	1	–	1.65	–	5.6	–	0.0503
BC	3.6	2.3	1	1	1.68	1.65	4.57	1.9	0.0558	0.2137
A^2^	− 4.9	− 4.2	1	1	1.63	1.61	9.14	6.8	0.0116	0.0347
B^2^	–	1.3	–	1	–	1.61	–	0.6	–	0.4603
C^2^	–	1.8	–	1	–	1.61	–	1.3	–	0.2915

The maximum removal of Cd^2+^ ions was achieved in run 6 with a value of 92.62% by the free *T. ornate* biomass, under an algal dosage of 6 g L^−1^, pH of 5, and initial Cd^2+^ concentration of 25 mg L^−1^, while the highest cadmium removal efficiency was obtained in run 10 with a value of 95.87% by the immobilized algal biomass, under an algal dosage of 4 g L^−1^, pH of 7, and Cd^2+^ ion concentration of 25 mg L^−1^.

To describe the correlation between the cadmium removal percentage and the tested factors, two second-order polynomial equations were established, and the final models for the freely suspended biomass (Eq. 1) and immobilized *T. ornata* biomass (Eq. 2) achieved through backward exclusion of insignificant factors in terms of the coded independent variables are expressed as follows:

1 $$\% \;{\text{Cd}}^{{{2} + }} \;{\text{removal}}\;{\text{efficiency}} = {87}.{31} + {5}.{82}A + {2}.{81}B - {4}.{81}C + {3}.{58}BC - {4}.{92}A^{{2}}$$

2 $$\begin{aligned} \% \;{\text{Cd}}^{{{2} + }} \;{\text{removal}}\;{\text{efficiency}} & = {86}.{84} + {7}.0{5}A + {2}.{79}B - {5}.{21}C{-}{1}.{85}AB \\ & \quad + \;{3}.{89}AC + {2}.{26}BC{-}{4}.{2}0A^{{2}} + {1}.{26}B^{{2}} + {1}.{83}C^{{2}} \\ \end{aligned}$$

where A, B, and C are the coded levels of the algal dose, pH and initial Cd^2+^ concentration, respectively.

Table S2 provides the ANOVA results for the quadratic regression model to determine the suitability and sufficiency of the predicted models. The *F* values (11.9 and 8.8 for the freely suspended and immobilized algae models, respectively) along with the low *p* value (< 0.005) suggested that the established models were highly statistically significant. The model terms were significant if *p* > *F* value was minimal (˂ 0.05). The lack-of-fit test was employed to assess model failure to clarify the experimental results within the experimental domain at stages not covered by regression analysis^25^. To indicate the model, this test must be nonsignificant. The lack of fit for both models was nonsignificant (*p* > 0.05), indicating that these models could adequately fit the experimental data.

Multiple regression analysis was performed to examine the results of BBD, as summarized in Table S2. A high determination coefficient value (*R*^2^ > 0.9) was found for both models via regression analysis, suggesting that the independent parameters were responsible for the majority of the variability. A regression model with a value of determination coefficient greater than 0.9 is supposed to achieve a strong correlation^26^. The experimental and predicted data notably agreed, where the adjusted values of *R*^2^ for the freely suspended and immobilized algal biomasses were high and near the expected values of *R*^2^ (Table S2).

The standard error of the estimation ratio to the mean value of the experimental response is referred to as the variance coefficient. According to Zar^27^, the higher the coefficient of variance value, the lower the experiment's reliability. Low values of the variance coefficient demonstrated the models' reliability and reproducibility (3.9% and 3.8% for the freely suspended and immobilized algal biomass models, respectively). The signal-to-noise ratio was calculated with an adequate accuracy, and the value was estimated through statistical analysis. According to Haaland^28^, a signal-to-noise ratio of 4 or higher is desirable and indicates appropriate model discrimination. The adequate precision values for the free and immobilized biomass models were 10.7 and 10.8, respectively, indicating that these models could be used for navigation of the design space. The model mean values of Cd^2+^ removal for the freely suspended and immobilized *T. ornata* biomasses were 84.9 and 86.3, respectively (Table S2).

### Analysis of the interactive impact of two variables on the Cd^2+^ removal efficiency

**Figure 2:**
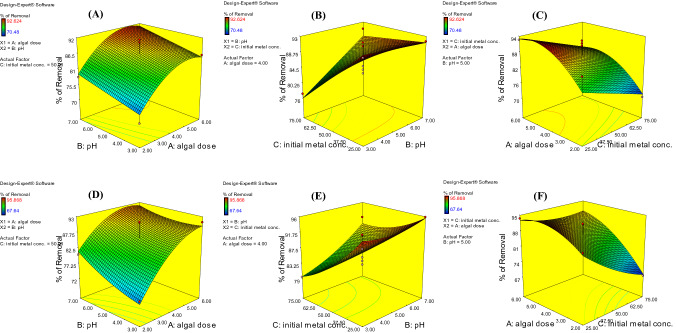
3-D response surface plots of Cd^2+^ ion biosorption using the freely suspended (**A**–**C**) and immobilized algal biomasses (**D**–**F**) under constant conditions: temperature 25 °C; time 90 min; shaking at 170 rpm.

Three-dimensional response surface plots and ANOVA findings include fundamental information on the influence of the investigated variables and aid in determining the type of relationship between these variables^29^. The effects of several variables, such as the algal dosage, pH, and initial Cd^2+^ concentration, on the Cd^2+^ removal efficiency for free and immobilized algal biomasses of *T. ornata* are shown in Fig. 2A–F. The figures were generated by combining two variables while keeping the third variable constant at the center point.

Figure 2A,D show the simultaneous effects of the algal dose and pH on Cd^2+^ removal. The efficiency of Cd^2+^ removal was improved by increasing the algal dose from 2 to 5 g L^−1^ and the pH from 3 to 7 for the free and Ca-alginate-immobilized algae.

The adsorbent dosage determines the amount of functional groups on the adsorbent surface in the adsorption process^30^. This parameter is considered the most significant factor controlling the heavy metal removal efficiency. Enhancement in elimination of metal ion at higher biomass concentrations could be attributed to a rise in the number of binding sites present on the cell surface, even as the amount adsorbed onto the adsorbent tended to decline^31^. These findings are supported by previous studies^32–34^.

The pH of the solution influences the dissociation state of the active functional groups on the adsorbent surface and metal ion speciation in aqueous solutions^35^. Raising the pH value from an acidic level to a neutral degree (3 to 7) improved the Cd^2+^ biosorption process by the free and immobilized *T. ornata* cells (Fig. 2A,D, respectively). The influence of the pH on the biosorption of Cd^2+^ ion by the freely suspended and immobilized *T. ornata* biomasses could be indicated by considering the chemistry of the algal surface, as evidenced by the estimated point of zero charge (pH_PZC_) with the Rivera–Utrilla et al. ^36^ method. The free and immobilized *T. ornata* cells had points of zero charge of 5.0 and 5.3, respectively. This suggests that a positive charge of the free and immobilized algal cell surfaces was maintained until pH levels of 5.0 and 5.3, respectively, after which the charge of these biosorbents became negative. As a result, the surfaces of the biosorbents were completely protonated at a low pH < pH_PZC_. This suggests that there exists electrostatic repulsion between cadmium ions and the surface of biosorbents, resulting in decreased Cd^2+^ biosorption onto the freely suspended and immobilized algal biomass surfaces. When the pH was raised to 7, there occurred a considerable decrease in the number of positively charged adsorption sites, which was confirmed by the continuous increase in the cadmium biosorption capacity of the biosorbents^37^.

Figure 2B,E show the simultaneous impacts of the pH and initial concentration of Cd^2+^ ions on Cd^2+^ elimination by the free and immobilized *T. ornata* cells, respectively. As shown in the diagram, an increase in the pH and decrease in the initial Cd^2+^ concentration caused an increase in Cd^2+^ removal. The resulting increase in accumulation of Cd^2+^ ion and reduction in the available biosorption sites in the algal biomass could explain the negative impact of the Cd^2+^ concentration on the elimination efficiency by the freely suspended and immobilized cells. At lower metal concentrations, all cadmium ions can interact with the available biosorption sites, resulting in a higher biosorption efficiency than at higher Cd^2+^ concentrations. At higher metal concentrations, more ions remain unadsorbed in the aqueous solution owing to saturation of the available biosorption sites. Similar results have also been stated in the literature^11,38^. It has been determined with increasing initial concentration, the efficiency of metal ion elimination decreased. This biosorption property revealed that surface saturation depended on the metal concentration^13^.

Figure 2C,F show the simultaneous effects of the algal dosage and initial Cd^2+^ concentration on Cd^2+^ removal by the free and Ca-alginate-immobilized algae, respectively. The percentage of Cd^2+^ removal increased with increasing algal dosage and decreasing initial Cd^2+^ concentration.

The obtained ANOVA data revealed that the algal dose was the most statistically significant variable affecting the Cd^2+^ removal efficiency (*p* = 0.0005), followed by the initial concentration of Cd^2+^ ions (*p* = 0.0019 and 0.0029) and pH (*p* = 0.037 and 0.047, respectively) for the freely suspended and immobilized *T. ornata* biomasses, respectively (Table 1).

The algal dose exerted a negative significant impact in quadratic terms for both the freely suspended and Ca-alginate-immobilized *T. ornata* cells. However, the algal dose attained a positive nonsignificant mutual relationship with the initial concentration of Cd^2+^ ions for the immobilized algal biomass (Table 1). Additionally, the nonsignificant, negative value for the interactive variables of the algal dose and pH for the immobilized algal biomass indicated that there existed no relationship between these variables, indicating that a change in one variable did not affect the other variable (*p* > 0.05; Table 1). The pH exerted a significant impact on the Cd^2+^ removal efficiency in linear terms only (*p* < 0.05; Table 1) for the freely suspended and immobilized biomasses. However, in the interactive term with the initial concentration of cadmium ions, the pH yielded a nonsignificant, positive impact for both the free and Ca-alginate immobilized algal biomasses.

### Model verification

The freely suspended and immobilized *Turbinaria ornata* biomasses were studied under optimal conditions to validate the expected results. Considering the desirability function criteria in Design Expert software, the optimum conditions for the independent variables were predicted. The maximum predicted removal efficiency for the free and immobilized algae could be attained under algal doses of 4.96 g L^−1^ and 5.04 g L^−1^, respectively, pH values of 6.84 and 5.06, respectively, and initial cadmium concentrations of 26.19 mg L^−1^ and 25.2 mg L^−1^, respectively. Triplicate experiments were carried out under these optimum conditions, with the mean values of the experimental data compared to the expected data. The experimentally observed values of the cadmium removal efficiency were 94.34% and 98.65% for the free and immobilized biomasses of *T. ornata*, respectively, matching well with expected values of 92.99% and 95.99%, respectively. The percentage prediction errors for the free and Ca-alginate-immobilized algae were 1.4% and 2.7%, respectively. As a result, the developed model was reliable and accurate for the prediction of the cadmium removal efficiency by freely suspended and immobilized *T. ornata* biomasses.

### Isotherm studies

By varying the initial concentrations of Cd^2+^ ions, isotherms were evaluated under a biosorbent dosage of 4 g L^−1^, pH of 5, and contact time of 90 min. To compare the experimental results and determine different isotherm parameters, the Langmuir, Freundlich, Temkin, and Dubinin–Radushkevich (D–R) isotherm models were applied.

**Figure 3:**
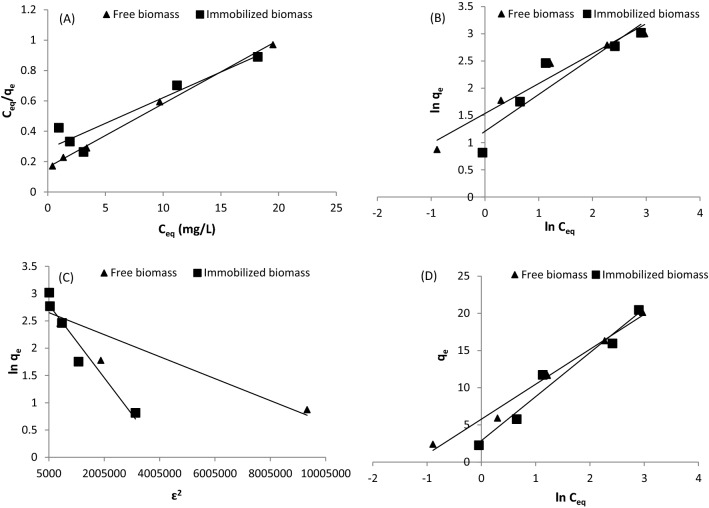
(**A**) Langmuir, (**B**) Freundlich, (**C**) Dubinin and Radushkevich, and (**D**) Temkin isotherm models of Cd^2+^ ion biosorption by the freely suspended and Ca-alginate-immobilized *T. ornata* cells (biosorbent dose = 4 g/L; pH = 5.0; contact time = 90 min; temperature 25 °C; shaking at 170 rpm).

According to the Langmuir isotherm model, adsorbate sorption occurs in monolayers at homogeneous sorption sites. Table 2 provides the main parameters determined with the Langmuir model at equilibrium. The maximum biosorption capacity of Cd^2+^ ions was 23.9 mg g^−1^ for the free algae and 29.6 mg g^−1^ for the immobilized biomass beads, which was greater than that of the freely suspended biomass, indicating that the immobilized algal biomass exhibited better Cd^2+^ biosorption properties than those of the free algal cells. These results were compared to those for other biosorbents reported in the literature^39^, showing that immobilized biomasses are suitable and effective biosorbents for the removal of cadmium and other heavy metals from aqueous solutions. The existence of alginate also improved the metal binding stability of the Cd^2+^ ion affinity over free algal cells, in which the biosorbent properties of the immobilized biomass differed from those of the freely suspended algae. The quantity of guluronic and other uronic acids in the alginate is assumed to be correlated to its affinity for heavy metals^40^. These acids are responsible for the removal of Cd^2+^ ions since they provide the majority of the carboxyl groups in alginate. The coefficient of determination values for Cd^2+^ ion removal with the freely suspended and alginate-immobilized *T. ornate* cells were higher than 0.9 (Fig. 3A, Table 2), indicating that the equilibrium results were well matched with the Langmuir isotherm model. The mechanism of heavy metal biosorption by algae may include monolayer coverage formation, ion exchange of Cd^2+^ ions or chemical biosorption^41^.

**Table 2:** Isotherm parameters for Cd^2+^ biosorption with the free and immobilized *T. ornata* biomasses.

Isotherms	Constants	Values	
		Free biomass	Immobilized biomass
Langmuir	*q*_*max*_ (mg g^−1^)	23.9	29.6
	*b* (L mg^−1^)	0.12	0.26
	*R*_*L*_	0.04–0.22	0.07–0.31
	*R*^2^	0.998	0.906
Freundlich	*n*	1.58	1.47
	*K*_*f*_ (L mg^−1^)	4.64	3.34
	*R*^2^	0.958	0.901
D–R	*q*_*o*_ (mg g^−1^)	14.2	16.4
	*β* × 10^−7^ (mol^2^ J^−2^)	2.0	7.0
	*E* (kJ mol^−1^)	15.81	8.45
	*R*^2^	0.861	0.946
Temkin	*A* (Lmg^−1^)	3.41	1.63
	*b* (J mol^−1^)	528.3	418.5
	*R*^2^	0.988	0.966

The favorability and form of the Cd^2+^ biosorption process can be identified with a dimensionless separation factor (*R*_*L*_), which can be calculated with Eq. (3):

3 $$R_{L} = 1/\left( {1 + bC_{0} } \right)$$

where *C*_*o*_ is the initial concentration of Cd^2+^ ions. The value of *R*_*L*_ shows whether the biosorption process is irreversible (*R*_*L*_ = 0), linear (*R*_*L*_ = 1), favorable (0 < *R*_*L*_ < 1) or undesirable (*R*_*L*_ > 1)^42^. The value of *R*_*L*_ for Cd^2+^ ions varied between 0.04 and 0.22 for the free algal cells and ranged from 0.07 to 0.31 for the immobilized biomass of *T. ornata*, suggesting a favorable biosorption process.

The Freundlich isotherm model supposes that the biosorbent exhibits a heterogeneous surface with a variety of binding sites. Figure 3B shows a plot of the Freundlich isotherm model for Cd^2+^ ion biosorption with the free and immobilized biomasses. As indicated in Table 2, the coefficient of determination (*R*^2^) values for the free algae and immobilized cells obtained with the Freundlich model are relatively smaller than those obtained with the Langmuir isotherm model. This suggests that the Langmuir model can fit the experimental results better than can the Freundlich isotherm model. Nevertheless, the biosorption intensity (*n*) and magnitude of the biosorption ability (*K*_*f*_) of the free and immobilized cells revealed easy Cd^2+^ ion uptake from the aqueous solutions with a high biosorption capacity. Moreover, the observed value of *1/n* varying between 1 and 10 for the Freundlich model indicated heterogeneity in the biosorbent surface and instantly revealed favorable Cd^2+^ ion biosorption onto both the free and Ca-alginate immobilized biomasses of *T. ornata*^43^.

The Dubinin–Radushkevich (D–R) adsorption model was employed to determine how pores are filled and to evaluate the physical or chemical nature of the biosorption process^44^.

The D–R model did not describe the experimental results achieved for the freely suspended algal biomass very well, since the coefficient of determination value was lower (*R*^2^ = 0.861; Fig. 3C) than that obtained for the immobilized algal biomass (*R*^2^ = 0.946). Additionally, the immobilized *T. ornata* biomass attained a relatively higher biosorption capacity of Cd^2+^ ions (*q*_*o*_ = 16.4 mg g^−1^) than that attained by the freely suspended algal cells (*q*_*o*_ = 14.2 mg g^−1^; Table 2). As a result, immobilization of *T. ornata* in alginate beads provided a greater ability to remove Cd^2+^ ions from aqueous solutions than that provided by the free algal cells.

The mean biosorption energy (*E*, kJ mol^−1^) reflects the physical or chemical biosorption process. If the *E* value is lower than 8 kJ mol^−1^, the biosorption process occurs physically, and if the value varies between 8 and 16 kJ mol^−1^, the process occurs chemically^45,46^. Since the *E* value was greater than 8 kJ mol^−1^ (Table 2), it was hypothesized that Cd^2+^ biosorption onto the free and immobilized *T. ornata* biomasses was accomplished via a chemisorption process.

According to the Temkin isotherm model, the adsorption heat of all molecules linearly increases with increasing coverage^47^. The intercept and slope of the linear curve of *q*_*e*_ against ln *C*_*eq*_ were considered to determine the constants of the Temkin model (*A* and *b*) (Fig. 3D). Regarding the freely suspended and immobilized algal cells, the *b* values were 528.3 and 418.5 J mol^−1^, respectively, suggesting a close association between the biosorbate and the surface of the biosorbent (Table 2). The Cd^2+^ biosorption results for the free and immobilized *T. ornata* algal biomasses were fitted with the Temkin isotherm model, yielding a high value of *R*^2^ (0.988 and 0.966, respectively).

### Kinetic studies

Adsorption kinetics are widely acknowledged for their utility in describing the metal uptake controlling the contact time of metal biosorption at the interface of solid/solution. As a result, the rate of adsorption for a given system is the most significant factor in adsorption system design.

The biosorption results were evaluated with different kinetic models: pseudo-first-order, pseudo-second-order, intra-particle diffusion, and Elovich models. Regarding various systems of biosorbents/biosorbates, pseudo-first- and pseudo-second-order kinetic models are the most commonly utilized models to assess the kinetic parameters of the Cd^2+^ biosorption process by free and Ca-alginate immobilized *T. ornata* biomasses. These models are the most basic models to express the relationship between biosorption of metal and the amount of available binding sites on the surface of the biosorbent^48^.

**Figure 4:**
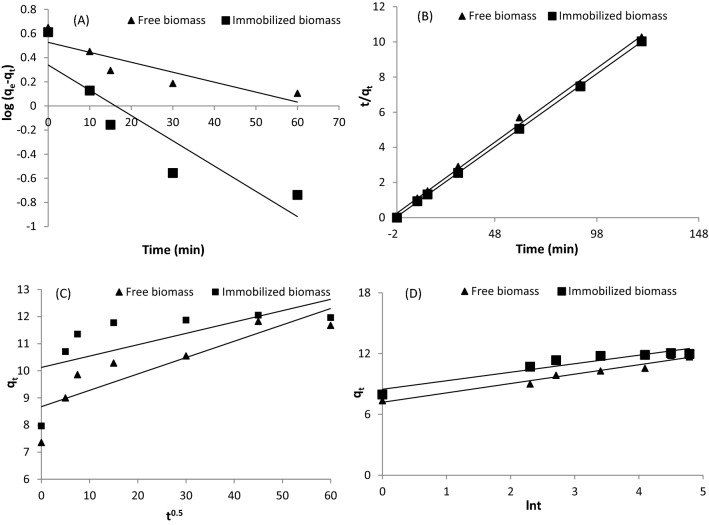
(**A**) Pseudo-first-order, (**B**) pseudo-second-order, (**C**) Intra-particle diffusion, and D) Elovich kinetic models of Cd^2+^ ion biosorption by the freely suspended and Ca-alginate-immobilized *T. ornata* cells (biosorbent dose = 4 g/L; pH = 5.0; initial Cd^2+^ concentration = 10 mg/L; temperature 25 °C; shaking at 170 rpm).

The intercept and slope of the curve of *ln*(*q*_*e*_- *q*_*t*_) against *t* were determined to quantify the kinetic parameters *q*_*e*_ and *k*_1_, respectively, of the pseudo-first-order model (Fig. 4A).

Furthermore, by plotting *t/q*_*t*_ against *t*, the parameters *k*_2_ and *q*_*e*_ of the pseudo-second-order model were calculated from the intercept and slope, respectively. Table 3 provides a comparison of the experimental biosorption capacities for the free and immobilized *T. ornata* biomasses and the kinetic constants obtained with the pseudo-first- and second-order kinetic models. For the freely suspended and immobilized *T. ornata* biomasses, the rate constants *k*_1_ for Cd^2+^ ions were estimated as 0.019 and 0.048 min^−1^, respectively. The immobilized algal biomass attained a higher kinetic rate (*k*_2_; 0.117 g mg^−1^ min^−1^) than that of the freely suspended algae, suggesting that the cation interaction process with the alginate beads occurred faster.

**Table 3:** Kinetic parameters for Cd^2+^ biosorption with the free and immobilized *T. ornata* biomasses.

Parameters	Values	
	Free biomass	Immobilized biomass
**Experimental data**		
*q*_*e*_ (exp.) (mg g^−1^)	12.05	
**Pseudo-first-order**		
*q*_*e*_ (cal.) (mg g^−1^)	3.37	2.18
*k*_1_ (min^−1^)	0.019	0.048
*R*^2^	0.783	0.819
**Pseudo-second-order**		
*q*_*e*_ (cal.)(mg g^−1^)	11.89	12.06
*k*_2_ (g mg^−1^ min^−1^)	0.027	0.117
*R*^2^	0.996	0.999
**Intra-particle diffusion**		
*K*_*i*_ (mg g^−1^ min^−0.5^)	0.06	0.042
*C*_*i*_ (mg g^−1^)	8.68	10.1
*R*^2^	0.769	0.421
**Elovich**		
*α* (g mg^−1^ min^−1^)	1.9E+44	5.7E+25
*β* (g mg^−1^)	1.08	1.19
*R*^2^	0.961	0.903

The coefficient of determination values obtained with the pseudo-first-order model were 0.783 and 0.819 for the freely suspended and Ca-alginate-immobilized biomasses, respectively.

The pseudo-second-order model for Cd^2+^ ion removal attained higher determination coefficient values (0.996 and 0.999; Fig. 4B, Table 3), indicating that this model captured the experimental results well for both the freely suspended and immobilized *T. ornata* biomasses, respectively, than did the pseudo-first-order model. Table 5 shows that the biosorption capacities calculated with the pseudo-second-order kinetic model for the free and alginate-immobilized *T. ornata* biomasses are closer to those estimated in the experiments than are those calculated with the pseudo-first-order models. As a result, the pseudo-first-order kinetic model is unsuitable to describe Cd^2+^ ion biosorption by freely suspended and immobilized algal cells, and biosorption follows pseudo-second-order model kinetics. In the Cd^2+^ biosorption process, the rate-limiting step is chemical sorption, which involves valance forces and the exchange or sharing of electrons between biosorbent functional groups and Cd^2+^ ions^49^.

The intra-particle diffusion model was utilized to describe the adsorption mechanism^50^. Figure 4C shows the data of linear regression of *q*_*t*_ against *t*^0.5^ for the free and immobilized algal cells. The linear plots in any of the cases did not pass through the origin (*C*_*i*_ > 0), indicating that intra-particle diffusion was not the only rate-limiting stage^51^ and that biosorption of Cd^2+^ ions by the freely suspended and immobilized algal biomasses was influenced by more than one mechanism^52^. These linear plots may be divided into multilinearity correlations, revealing that three steps occurred throughout the entire Cd^2+^ biosorption process involving the free and immobilized *T. ornata* biomasses^53^.

The first linear part can be ascribed to the transfer of Cd^2+^ ions from the solution to the external surface of the free and immobilized cells via film flow or the diffusion of Cd^2+^ ions through the boundary layer^54^. At this stage, the transfer process of Cd^2+^ ions for the immobilized biomass was quicker than that for the free cells, which is likely attributable to the greater surface area and larger number of carboxylate groups of the Ca-alginate-immobilized *T. ornata* biomass than those of the free biomass. Particularly, the influence of carboxylate groups might yield a more hydrophilic biosorbent surface, thereby increasing Cd^2+^ transfer from the solution to the external surface of immobilized biomass, resulting in more notable biosorption onto the immobilized *T. ornata* biomass than that onto the free algal cells. The second linear part indicates progressive biosorption, where intra-particle diffusion is the rate-controlling step. The third part is ascribed to the final equilibrium where the process of intra-particle diffusion begins to decelerate owing to the very low ion concentrations remaining in solution.

As a result, external diffusion through films largely controls the Cd^2+^ biosorption mechanism onto biosorbents, followed by intra-particle diffusion.

The intercept was high (*C*_*i*_, 8.6 and 10.1 mg/g for the freely suspended and immobilized *T. ornata* biomasses, respectively), which might be attributable to an increased boundary layer thickness, reduced external mass transfer and increased internal mass transfer^45^.

The Elovich model suggests that active binding sites are heterogeneous, with different adsorption energies^55^. The high determination coefficients values (0.961 and 0.903 for the freely suspended and immobilized *T. ornata* cells, respectively) indicate that the kinetic results follow the Elovich kinetic model (Table 3; Fig. 4D). Moreover, higher Elovich parameter values (β and α) determined from the slope and intercept, respectively, of the curve of *q*_*t*_ against *lnt*, indicate a higher rate of chemical sorption confirming the adsorption type of a pseudo-second-order^52^.

### Thermodynamic studies

To demonstrate the thermodynamic behavior of the Cd^2+^ ion biosorption process, thermodynamic parameters such as the Gibbs free energy change (*ΔG*^*o*^), entropy (*ΔS*^*o*^) and enthalpy (Δ*H*^*o*^) change can be determined. Table 4 lists the changes in entropy and enthalpy calculated from the intercept and slope, respectively, of the curve of *ln K* against 1/*T*.

**Table 4:** Thermodynamic parameters for Cd^2+^ biosorption with the free and immobilized *T. ornata* biomasses.

Temperature (K)	*ΔG*^*o*^ (kJ mol^−1^)		*ΔH*^*o*^ (kJ mol^−1^)		*ΔS*^*o*^ (kJ mol^−1^)		*R*^2^	
	Free biomass	Immob. biomass	Free biomass	Immob. biomass	Free biomass	Immob. biomass	Free biomass	Immob. biomass
298	− 2.24	− 3.06	67.5	19.2	0.234	0.075	0.995	0.966
308	− 4.33	− 3.61						
318	− 6.93	− 4.57						

*ΔG*^*o*^ measures the spontaneity of the biosorption process, with a higher negative value indicating more notably favorable biosorption. The thermodynamic feasibility and spontaneous nature of the Cd^2+^ biosorption process onto the free and alginate-immobilized *T. ornata* biomasses were confirmed by the obtained negative *ΔG*^*o*^ values^44^. The values of *ΔG*^*o*^ for the free and immobilized *T. ornata* biomasses decreased from − 2.24 to − 6.93 kJ mol^−1^ and − 3.06 to − 4.57 kJ mol^−1^, respectively, as the temperature was increased from 298 to 318 K (Table 4; Fig. S1). The decline in the values of *ΔG*^*o*^ with increasing temperature shows that biosorption is  more feasible at higher temperatures. Copper biosorption from aqueous solutions by *Codium vermilara* exhibits a similar pattern^15^.

The biosorption process of Cd^2+^ ions onto the free and immobilized cells was endothermic, as evidenced by the positive *ΔH*^*o*^ values. Furthermore, the improved randomness during Cd^2+^ biosorption at the interface of solid/solution was indicated by the determined positive *ΔS*^*o*^ values (Table 4)^56^.

At varying temperatures, the biosorption efficiency of each algal species varies with each metal ion^13^. Certain results have indicated that metal biosorption by algae is endothermic in nature and that the biosorption efficiency improves with increasing temperature^11^. Nevertheless, the temperature exerts a negligible impact on metal biosorption by algae, according to several studies, and other studies have examined temperature-related differences in biosorption of heavy metals ability by algae^57^. These seemingly contradictory findings can be explained by the fact that optimum temperatures for biological reactions in algae are usually constrained within a limited range and that temperature changes can cause various biosorption behaviors in several algae when exposed to different metal ions.

### Characterization of the *T. ornata* biomass

#### Scanning electron microscopy (SEM) and energy-dispersive X-ray analysis (EDX) studies

**Figure 5:**
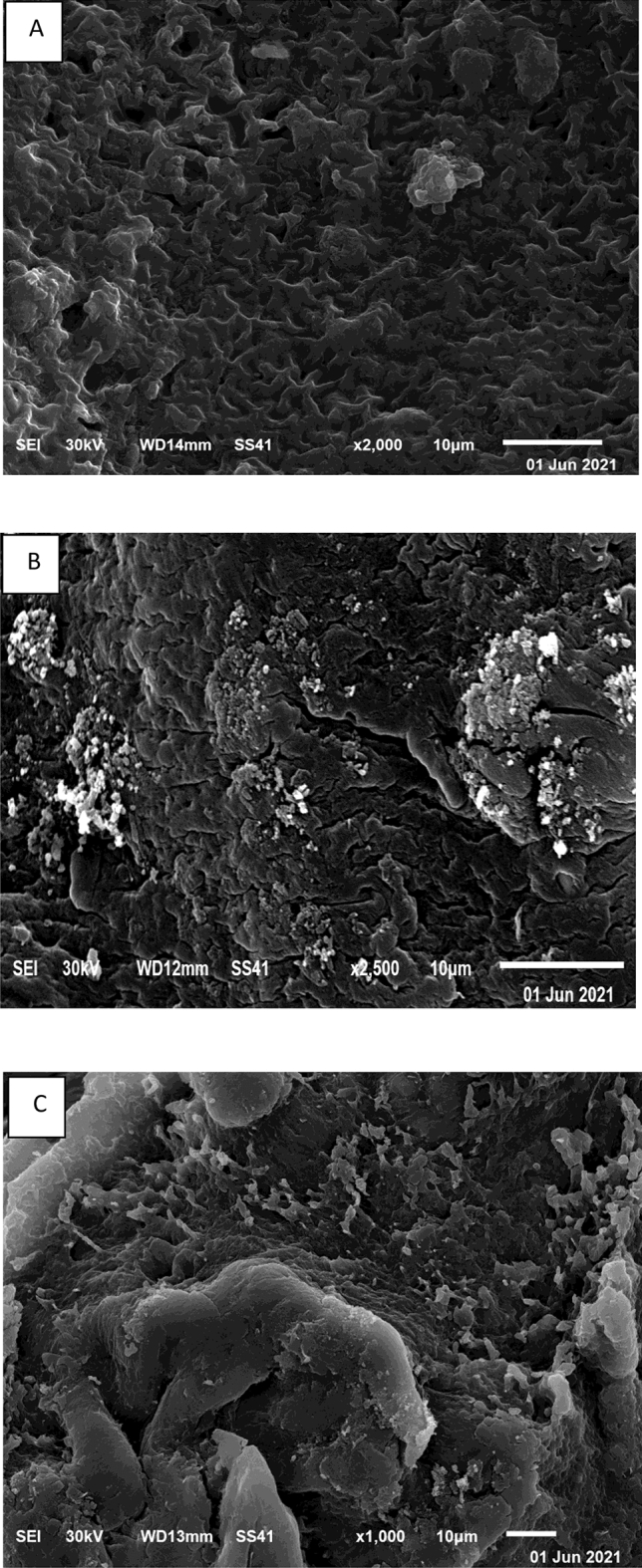
SEM micrographs of the algal biomass before (**A**) and after Cd^2+^ biosorption for the freely suspended (**B**) and Ca-alginate-immobilized *T. ornata* cells (**C**).

**Figure 6:**
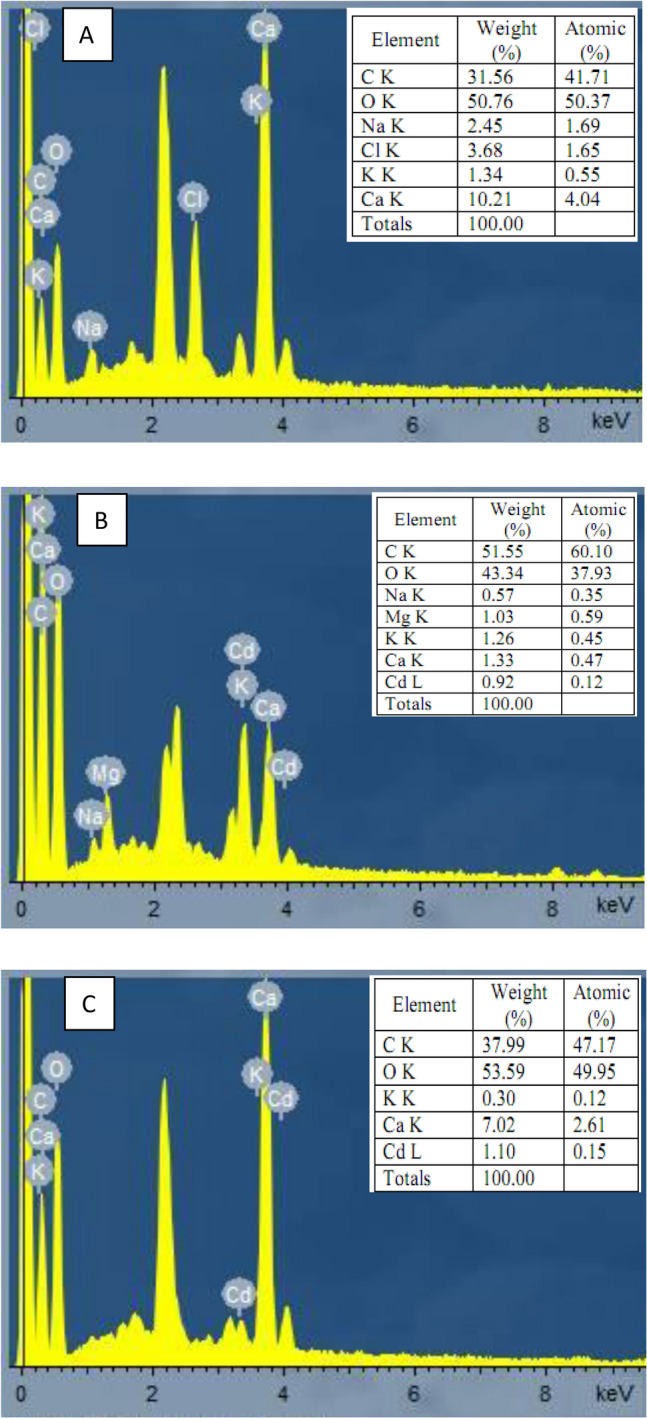
EDX micrographs of the algal biomass before (**A**) and after Cd^2+^ biosorption for the freely suspended (**B**) and Ca-alginate-immobilized *T. ornata* cells (**C**).

The morphology of the algal biomass before Cd^2+^ biosorption as well as of the freely suspended and Ca-alginate immobilized cells after biosorption was characterized via SEM–EDX (Figs. 5, 6, respectively). Figure 5A shows that *T. ornata* exhibits a very rough surface with a large number of pores, indicating that there exists a considerable potential for cadmium ion biosorption. A reduction in the pore number and discrete lump formation across the surface of the free and immobilized algal biomasses occurred after Cd^2+^ biosorption (Fig. 5B,C, respectively), suggesting cadmium sorption onto the surface of the biosorbent. Additionally, as a result of cadmium ion accumulation on the cell surface, the surface of the free and immobilized *T. ornata* biomasses decreased, became more irregular, and exhibited spots. These morphological alterations confirmed the interaction of cadmium ions with surface functional groups provided by the freely suspended cells and cross-linked beads^58^.

EDX analysis was conducted to provide information on the elemental composition of the surface of the algal biomass and to verify the existence of cadmium ions bound to the surface of the freely suspended and immobilized algal biomasses^59^. The results verified the existence of carbon and oxygen, which are the major components of cellular macromolecules (Fig. 6A–C). Calcium, sodium and potassium ions were the major cations that were exchanged throughout the biosorption process. As a result, the signal intensity of these cation peaks could decrease or perhaps disappear (Fig. 6B,C). This suggests that ion exchange was the mechanism responsible for Cd^2+^ ion biosorption. A relatively higher peak area relating to Cd^2+^ was observed for the Ca-alginate immobilized algal cells after cadmium biosorption than that observed for the free algae (Fig. 6B,C, respectively), proposing a higher biosorption capacity of the immobilized cells. El-Naggar et al.^60^ found that when the biomass of *Ulva fasciata* was exposed to cadmium, the typical peak of cadmium emerged. These findings are similar to those of Huang et al.^61^ and Saleh et al.^62^.

#### Fourier transform infrared (FTIR) analysis

The cell walls of algae consist of several functional groups, including phosphate, sulfate, amine, carboxyl and hydroxyl groups, which are important in the binding process of metal ions^63^. To study the interaction of Cd^2+^ ions with the functional groups of algal cell walls, the spectra of the free and immobilized algal biomasses after biosorption were compared to those of the corresponding pure algal biomasses (Fig. S2A–C). Biosorption of heavy metals onto the cell surface occurs via the ion exchange mechanism. The high peak observed for the pure algal biomass at 3424 cm^−1^, representing the stretching of the O–H and N–H bonds of the amine groups, shifted after Cd biosorption for the freely and immobilized algal cells, suggesting that the bonds of O–H and N–H are involved in Cd^2+^ ion biosorption^64^. The band at 2924 cm^−1^ represents C–H bonds, showing the presence of carboxylic groups from fractions of phospholipids and lipids in the *T. ornata* biomass^65^. Carboxylic and amino groups account for 70% of all cell wall functional groups in most brown algae, and these groups are important for the binding process of metal ions^66^. Peaks at 1745 and 1521 cm^−1^ were only observed for the pure biomass and after Cd^2+^ absorption for the immobilized algal biomass, suggesting that NO_2_, C = O, and S groups are involved in the biosorption process of cadmium^67^. The absorption peaks at 1626, 1627 and 1628 cm^−1^ in all treatments were related to the stretching vibration of C=N. The bands at 1462 cm^−1^ shifted to 1425 and 1460 cm^−1^ after biosorption of Cd^2+^ in the spectra of the free and immobilized algae, respectively, and were related to CH_2_ and CH_3_ bending of methyl groups and C–O stretching vibration of carboxylic groups. Asymmetric stretching vibration of phosphodiester P=O was responsible for the protein band detected only at 1240 cm^−1^ in the spectra of the immobilized algal surfaces treated with cadmium ions^68^. The peaks at 1266 and 1274 cm^−1^ present only after biosorption of Cd ions in the spectra of the free and immobilized algal biomasses, respectively, were assigned to the stretching vibrations of C–C–O, while those at 1162 and 1159 cm^−1^ represented the stretching vibrations of hydrogen–bonding C–OH groups, implying the presence of carbohydrates in the algal samples^13^. In regard to the pure biomass, the band at 1045 cm^−1^, which indicates the stretching vibrations of C–O (primary alcohol), shifted to 1032 and 1039 cm^−1^ after Cd^2+^ biosorption in the spectra of the free and Ca-alginate immobilized cells, respectively. Furthermore, the shift in the band at 895 cm^−1^ for the pure biomass to 822 and 870 cm^−1^ after cadmium biosorption for the free and immobilized algal cells, respectively, revealed cadmium ion bonding to the amine group occurring on the algal surface. Alterations in the vibrational frequency of the cell wall functional groups following biosorption of Cd^2+^ ions demonstrated that these groups were involved in biosorption.

### Comparison to other biosorbents

Table 5 provides a comparison of the removal efficiency (percentage) of Cd^2+^ ions in this study to that determined in other studies published in the literature^14,60,69–74^. The Cd^2+^ elimination efficiency achieved in this study was much higher than that described for other biosorbents. As a result, freely suspended and immobilized *Turbinaria ornata* biomasses are excellent biosorbents for the removal of cadmium ions from aqueous solutions.

**Table 5:** Maximum removal efficiency of Cd^2+^ ions by low-cost biosorbents.

Biosorbent	Cd^2+^ removal efficiency (%)	References
*Merismopedia tenuissima*	66.7	^14^
Immobilized *Ulva fasciata* biomass	98.19	^60^
*Chlorella vulgaris*	89.0	^69^
Immobilized cells of *Scenedesmus quadricauda*	90.0	^70^
*Hypnea valentiae*	86.8	^71^
*Pseudochlorococcum typicum*	86.0	^72^
*Ulva compressa*	94.8	^73^
*Trichormus variabilis*	89.0	^74^
*Turbinaria ornata*	94.34	Present study
Immobilized *Turbinaria ornata* biomass	98.65	Present study

## Conclusion

Marine alga *T. ornata* biomass was immobilized with Ca-alginate, and the Cd^2+^ ion removal efficiency was assessed. The most significant conditions to attain the maximum Cd^2+^ biosorption from aqueous solutions using free and alginate-immobilized *T. ornata* biomasses were identified with the Box–Behnken design method. The conditions to achieve the maximum cadmium biosorption with free and immobilized cells were determined as algal doses of 4.96 g L^−1^ and 5.04 g L^−1^, pH values of 6.84 and 5.06, and initial cadmium concentrations of 26.19 mg L^−1^ and 25.2 mg L^−1^, respectively. Under these optimal conditions, the free and immobilized *T. ornata* biomasses attained maximal cadmium removal efficiencies of 94.34% and 98.65%, respectively. The Langmuir, Freundlich, and Temkin sorption isotherm models as well as pseudo-second-order and Elovich kinetic models could best fit the experimental results for cadmium ion removal with the freely suspended and immobilized algal biomasses. The thermodynamic parameters revealed that the cadmium ion biosorption process was spontaneous, feasible, and endothermic in nature. SEM, EDX and FTIR analyses showed notable alterations in the characteristics of the free and immobilized algae as a result of the Cd^2+^ biosorption process. In conclusion, it was confirmed that *T. ornata* biomass immobilized with Ca-alginate could be employed to efficiently remove Cd^2+^ ions from aqueous solutions.

## Materials and methods

### Algal material and preparation of the cadmium solution

*Turbinaria ornata* (Turner) J. Agardh, a marine brown alga, was collected from the Red Sea coast in Jeddah, Saudi Arabia. The alga was vigorously washed with tap water to remove salts and epiphytic microorganisms, washed with distilled water, dried to a constant weight in an oven at 40 °C, ground, and sieved into 125-µm fractions.

Cd^2+^ solutions of different concentrations were obtained by diluting a 1000 mg L^−1^ Cd^2+^ stock solution made by dissolving suitable quantities of CdSO_4_.8/3H_2_O (99.0% purity, Sigma-Aldrich) in 1000 mL of deionized water.

### Immobilization of the algal biomass

The algal biomass was immobilized by dissolving 4 g of Na-alginate in 100 mL distilled water for 30 min at 60 °C under constant stirring^75^. Following cooling, 4 g of dried *T. ornata* biomass was added to the solution of sodium alginate and stirred for 5 min at room temperature. This mixture of the alginate-algal biomass was dropped into a 2% cold solution of CaCl_2_ with a 3 mL syringe to obtain 1.5 ± 0.2 mm diameter beads. To enhance the mechanical strength of the produced beads, they were soaked in a solution of calcium chloride for 2 h at room temperature under gentle stirring. For removing any residual Ca^2+^ from the bead surfaces, the beads were washed three times with distilled water and then placed in a refrigerator at 4 °C before application in the experiments.

### Biosorption experiments

The biosorption experiments were carried out in 250-mL conical flasks containing 100 mL of the above prepared Cd^2+^ ion solution at 25 °C. In order to investigate the influence of the initial Cd^2+^ concentration on the removal of Cd^2+^ ions, approximately 4 g L^−1^ freely suspended and immobilized biomasses was added to solutions with various cadmium concentrations (10, 25, 50, 75, and 100 mg L^−1^) at 90 min, pH of 5.0 and 170 rpm. The impact of the contact time on Cd^2+^ ion uptake was also investigated under the same conditions. At time intervals of 0, 10, 15, 30, 60, 90, 120, 150, and 180 min, filtrates were withdrawn.

The pH was adjusted with 0.1 M H_2_SO_4_ and 0.1 M NaOH. The algal biomass was removed from the aqueous solutions via centrifugation at 4000 rpm for 5 min. The concentration of the remaining Cd^2+^ ions in the solutions was determined with an inductively coupled plasma optical emission spectrometry (ICP–OES) instrument, Perkin Elmer Optima 2000DV.

The quantity of biosorbed Cd^2+^ at equilibrium (*q*_*e*_; mg g^−1^) was determined with Eq. (4):

4 $$q_{e} = \frac{{V \left( {C_{i} - C_{eq} } \right) }}{W}$$

where *C*_*i*_ and *C*_*eq*_ (mg L^−1^) are the initial and equilibrium Cd^2+^ concentrations, respectively, *W* (g) is the biosorbent mass, and *V* (mL) is the sample solution volume.

The removal efficiency of Cd^2+^ ions (*RE* %) by the *T. ornata* biomass was expressed as Eq. (5):

5 $${\text{Removal}}\;{\text{efficiency}}\left( {\text{\%}}\right) = \frac{{\left( {C_{i} - C_{eq} } \right) }}{{C_{i} }} \times 100.$$

All the determinations of cadmium ions in the aqueous solution were performed in triplicate.

### Experimental design

The response surface method (RSM) was employed to assess the impact of various factors on the cadmium elimination efficiency. The Design Expert statistical program version 7.0.0 was employed to statistically analyze the experimental design (Stat-Ease Inc., Minneapolis, USA). A three-factor Box**–**Behnken Design (BBD, a type of RSM) was employed to study and maximize the impact of three independent parameters, the algal dosage (2–6 g L^−1^), pH (3–7) and initial Cd^2+^ concentration (25–75 mg L^−1^), on the Cd^2+^ removal efficiency of the freely suspended and immobilized biomasses as well as to examine the interaction between these parameters (Table S1). The temperature was kept constant at 25 °C, the contact time was 90 min, and shaking occurred at 170 rpm during the experiments. The experiments were carried out according to the BBD method, with a total of 17 experiments and 5 center point replicates used to evaluate the pure error. The concentration of the remaining Cd^2+^ ions was determined as described previously.

Equation (6) can be used to represent the second-order polynomial model under the expected optimum conditions:

6 $${\text{Y}} = \upbeta_{{\text{o}}} + \sum \upbeta_{{\text{i}}} {\text{X}}_{{\text{i}}} + \sum \upbeta_{{{\text{ii}}}} {\text{X}}_{{\text{i}}}^{2} + \sum \upbeta_{{{\text{ij}}}} {\text{X}}_{{\text{i}}} {\text{X}}_{{\text{j}}} + \upvarepsilon$$

where Y denotes the predicted response, β_o_ denotes the intercept term, β_i_, β_ii_ and β_ij_ denote the linear, quadratic, and interaction influences, respectively, X_i_ and X_j_ are the independent factors, and ε is the error.

### Statistical analysis

Three-dimensional surface plots and ANOVA were employed to describe the relationships between the responses and independent factors, as well as to assess the optimal conditions for Cd^2+^ removal, and the significance was evaluated at certain probability levels via the *F* test (*p* ≤ 0.05).

### Isotherm studies

Biosorption isotherms describe the relationship between the biosorption capacity (*q*_*e*_) and the concentration of metal (*C*_*eq*_) at equilibrium. To characterize the biosorption process of the Cd^2+^ isotherm, Langmuir, Freundlich, Dubinin–Radushkevich (D–R), and Temkin isotherm models were used. The following is a list of the various model forms:

Langmuir model:

7 $$\frac{{C_{eq} }}{{q_{e} }} = \frac{1}{{q_{max} b}} + \frac{{C_{eq} }}{{q_{max} }}.$$

Freundlich model:

8 $$Ln \,q_{e} = Ln\, K_{f} + \frac{1}{n} Ln \,C_{eq} .$$

D–R model:

9 $$Lnq_{e} = lnq_{0} - \beta \varepsilon^{2}$$

10 $$\varepsilon = RT\left( {1 + \frac{1}{{C_{eq} }}} \right)$$

11 $$E = \sqrt {1/2} \beta .$$

Temkin model

12 $$q_{e} = B\,ln\,A + B\, ln\,C_{eq}$$

13 $$B = \frac{RT}{b}$$

where *q*_*max*_ (mg g^−1^) is the maximum biosorption capacity required for monolayer formation, *b* (L mg^−1^) is the Langmuir constant, *n* and *K*_*f*_ are the parameters of the Freundlich model, *E* (kJ mol^−1^) is the mean free energy of biosorption for the Dubinin–Radushkevich model, *q*_*o*_ (mg g^−1^) is the theoretical maximum biosorption capacity, *β* (mol^2^ J^−2^) is the constant of the D-R isotherm, *ε* (J mol^−1^) is the Polanyi potential corresponding to the equilibrium concentration, *R* (8.314 kJ mol^−1^) is the universal gas constant, *T* is the absolute temperature (K), *A* (L mg^−1^) is the equilibrium binding constant related to the maximum binding energy, and *b* (J mol^−1^) is the Temkin model constant corresponding to the sorption heat.

### Kinetics studies

The experimental data were presented using pseudo-first-order, pseudo-second-order, intra-particle diffusion, and Elovich kinetic models to better explain the biosorption dynamics. According to the following Eqs. (14–17), each model is represented as a linear form:

Pseudo-first-order model:

14 $$Log\, \left( {q_{e} - q_{t} } \right) = Log \,q_{e} - \frac{{K_{1} t}}{2.303}.$$

Pseudo-second-order model:

15 $$\frac{t}{{q_{t} }} = \frac{1}{{K_{2} q_{e}^{2} }} + \frac{t}{{q_{e} }}.$$

Intra-particle diffusion model:

16 $$q_{t} = K_{i} t^{1/2 } + C_{i} .$$

Elovich model:

17 $$q_{t} = \frac{1}{\beta }\ln \left( {\upalpha \upbeta } \right) + \frac{1}{\beta }\ln \left( {\text{t}} \right)$$

where *q*_*t*_ and *q*_*e*_ are the quantities of Cd^2+^ biosoption capacities (mg g^−1^) at time *t* and at equilibrium, respectively, *k*_*1*_ (min^−1^) and *k*_*2*_ (g mg^−1^ min^−1^) are the rate constants of the pseudo-first- and pseudo-second-order models, respectively, *K*_*i*_ (mg g^−1^ min^−0.5^) is the intra-particle diffusion rate constant, *C*_*i*_ (mg g^−1^) is the effect of boundary layer diffusion, α (g mg^−1^ min^−1^) is the initial sorption rate, and *β* (g mg^−1^) is the Elovich constant related to the extent of surface coverage and activation energy for chemisorption.

### Thermodynamics studies

Thermodynamic parameters could be assessed to explore the Cd^2+^ biosorption nature. The following equations were considered to determine the change in Gibbs free energy (*ΔG*^*o*^; kJ mol^−1^), change in entropy (*ΔS*^*o*^; kJ mol^−1^) and change in enthalpy (*ΔH*^*o*^; kJ mol^−1^)^76^:

18 $$\Delta G^{{\text{o}}} = - RTlnK_{C}$$

19 $$LnK_{C} = \frac{{{\Delta }S}}{R} - \frac{{{\Delta }H}}{RT}$$

20 $$\Delta G^{{\text{o}}} = \Delta H^{{\text{o}}} - T \Delta S^{{\text{o}}}$$

where *K*_*c*_ is the constant of thermodynamic equilibrium.

### Biosorbent characterization

Dried algal biomass before Cd^2+^ biosorption as well as freely suspended and Ca-alginate immobilized cells after biosorption were treated with a thin film of graphite followed by gold to improve the image resolution and analyzed with a scanning electron microscopy (SEM) instrument (JEOL JSM-6510 L. V operated at 30 kV). In addition, the algal biomasses were analyzed via energy-dispersive X-ray (EDX) analysis (JEOL JEM-2100 (HRTEM) operated at a voltage of 200 kV) connected to an SEM instrument at the Faculty of Science, Mansoura University, Egypt. This method was applied to determine the elements occurring on the wall as well as the mechanisms involved in the process of Cd^2+^ biosorption. The algal biomasses were further observed via Fourier transform infrared spectroscopy (FT-IR) (Thermo Fisher Scientific model FT-IR IS 10, USA) at the Faculty of Science, Mansoura University, Egypt. Infrared spectra in the range from 4000 to 500 cm^−1^ were obtained at a resolution of 4 cm^−1^.

All methods were performed in accordance with relevant guidelines and regulations.

## Supplementary Information

Supplementary Information.
