# Supplementary material for: Process optimization and modeling of Cd2+ biosorption onto the free and immobilized Turbinaria ornata using Box–Behnken experimental design
Source: Sci Rep. 2022 Feb 28;12:3256. doi: 10.1038/s41598-022-07288-z (PMC8885682; doi:10.1038/s41598-022-07288-z)
Supplement: Supplementary file 1 — Supplementary Information. [file 41598_2022_7288_MOESM1_ESM.docx]

**Supplementary Materials**

**Process optimization and modeling of Cd^2+^ biosorption onto the free and immobilized *Turbinaria ornata* using Box–Behnken experimental design**

**Mustafa A. Fawzy^1*^, Hadeer Darwish^2^, Sarah Alharthi^3^, Mayasar I. Al-Zaban^4*^, Ahmed Noureldeen^1^, Sedky H.A. Hassan^5, 6^**

^1^Biology Department, Faculty of Science, Taif University, P.O. Box 11099, Taif 21944, Saudi Arabia

^2^Biotechnology Department, Faculty of Science, Taif University, P.O. Box 11099, Taif 21944, Saudi Arabia

^3^Chemistry Department, Faculty of Science, Taif University, P.O. Box 11099 Taif, 21944, Saudi Arabia

^4^Biology Department, Faculty of Science, Princess Nourah Bint Abdulrahman University, Riyadh 11671, Saudi Arabia

^5^Department of Biology, College of Science, Sultan Qaboos University, Muscat 123, Oman

^6^Department of Botany and Microbiology, Faculty of Science, New Valley University, 72511, El-Kharga, Egypt

^1*^Corresponding author; email: [mafawzy@tu.edu.sa](mailto:mafawzy@tu.edu.sa); phone (+9665594732248);

^4*^Corresponding author; email: [mialzaban@pnu.edu.sa](mailto:mialzaban@pun.edu.sa); phone (+966555288787)

**Table S1. Box–Behnken experimental design with coded and uncoded operational variables (Factors A, B and C) and results for the Cd^2+^ removal efficiency (%) with the freely suspended and immobilized *T. ornata* biomasses**

| **Std. order** | **Factor A:**  **Algal dose (g/L)** | **Factor B: pH** | **Factor**  **C: Initial Cd^2+^ conc. (mg/L)** | **Freely suspended biomass** | | **Immobilized biomass** | |
| --- | --- | --- | --- | --- | --- | --- | --- |
|  |  |  |  | **Actual response:**  **RE^a^ (%)** | **Predicted response: RE^a^ (%)** | **Actual response:**  **RE^a^ (%)** | **Predicted response: RE^a^ (%)** |
| 1 | 2(-1) | 3(-1) | 50(0) | 70.81 | 73.76 | 73.08 | 72.20 |
| 2 | 6(+1) | 3(-1) | 50(0) | 86.05 | 85.40 | 91.14 | 90.00 |
| 3 | 2(-1) | 7(+1) | 50(0) | 78.24 | 79.38 | 80.35 | 81.50 |
| 4 | 6(+1) | 7(+1) | 50(0) | 88.08 | 91.02 | 91.00 | 91.88 |
| 5 | 2(-1) | 5(0) | 25(-1) | 86.76 | 81.38 | 87.48 | 86.53 |
| 6 | 6(+1) | 5(0) | 25(-1) | 92.62 | 93.02 | 93.52 | 92.83 |
| 7 | 2(-1) | 5(0) | 75(+1) | 70.48 | 71.76 | 67.64 | 68.33 |
| 8 | 6(+1) | 5(0) | 75(+1) | 86.09 | 83.40 | 89.25 | 90.20 |
| 9 | 4(0) | 3(-1) | 25(-1) | 92.28 | 92.90 | 92.77 | 94.60 |
| 10 | 4(0) | 7(+1) | 25(-1) | 91.62 | 91.35 | 95.87 | 95.67 |
| 11 | 4(0) | 3(-1) | 75(+1) | 77.28 | 76.11 | 79.48 | 79.67 |
| 12 | 4(0) | 7(+1) | 75(+1) | 90.94 | 88.89 | 91.60 | 89.77 |
| 13-17^b^ | 4(0) | 5(0) | 50(0) | 90.37 | 87.31 | 91.00 | 86.84 |

^a^ RE: Removal efficiency.

^b^ The mean value of five center-point assays.

**Table S2. Results of ANOVA for the response surface model of Cd^2+^ ion removal**

| **Source** | | **Squares sum** | | | | **Degrees of freedom** | | | | **Mean sum of squares** | | | | ***F* value** | | | | ***p* value**  **Prob > *F*** | | |
| --- | --- | --- | --- | --- | --- | --- | --- | --- | --- | --- | --- | --- | --- | --- | --- | --- | --- | --- | --- | --- |
|  | | **Free cells** | | **Immob. cells** | | **Free cells** | | **Immob. cells** | | **Free cells** | | **Immob. cells** | | **Free cells** | | **Immob. cells** | | **Free cells** | | **Immob. cells** |
| **Model** | | 673.1 | | 861.9 | | 5 | | 9 | | 134.6 | | 95.8 | | 11.99 | | 8.8 | | 0.0004* | | 0.0045* |
| **Residual** | | 123.5 | | 76.2 | | 11 | | 7 | | 11.2 | | 10.9 | | - | | - | | - | | - |
| **Lack of Fit** | | 64.8 | | 13.6 | | 7 | | 3 | | 9.3 | | 4.5 | | 0.63 | | 0.29 | | 0.721** | | 0.83** |
| **Pure Error** | | 58.7 | | 62.5 | | 4 | | 4 | | 14.7 | | 15.6 | | - | | - | | - | | - |
| **Correlation Total** | | 796.6 | | 938.0 | | 16 | | 16 | | - | | - | | - | | - | | - | | - |
| **R^2^** | | | **Adj. R^2^** | | | | **Pred. R^2^** | | | | **Adeq. precision** | | | | **Variation coefﬁcient %** | | | | **Mean** | |
| **Free cells** | **Immob. cells** | | **Free cells** | | **Immob. cells** | | **Free cells** | | **Immob. Cells** | | **Free cells** | | **Immob. Cells** | | **Free cells** | | **Immob. Cells** | | **Free cells** | **Immob. cells** |
| 0.91 | 0.93 | | 0.81 | | 0.86 | | 0.71 | | 0.72 | | 10.7 | | 10.8 | | 3.9 | | 3.8 | | 84.9 | 86.3 |

*Significant at p ˂ 0.05; **not significant at p> 0.05, Immob. Cells: immobilized *T. ornata* cells

**Fig. S1. Ln*Kc* plot versus 1/*T* for Cd^2+^ ion biosorption with the freely suspended and Ca-alginate-immobilized *T.* *ornata* cells (biosorbent dose= 4 g/L; pH= 5.0; initial Cd^2+^ concentration= 10 mg/L; contact time= 90 min; shaking at 170 rpm).**

(A)

(B)

(C)

**Fig.** **S2. FT-IR spectra of** **the algal biomass before (A) and after Cd^2+^ biosorption for the (B) freely suspended and (C) Ca-alginate immobilized *T.* *ornata* cells.**
